# Cancer Associated Fibroblasts in Stage I-IIIA NSCLC: Prognostic Impact and Their Correlations with Tumor Molecular Markers

**DOI:** 10.1371/journal.pone.0134965

**Published:** 2015-08-07

**Authors:** Thomas K. Kilvaer, Mehrdad Rakaee Khanehkenari, Turid Hellevik, Samer Al-Saad, Erna-Elise Paulsen, Roy M. Bremnes, Lill-Tove Busund, Tom Donnem, Inigo Z. Martinez

**Affiliations:** 1 Department of Oncology, University Hospital of North Norway, Tromso, Norway; 2 Institute of Clinical Medicine, UiT The Arctic University of Norway, Tromso, Norway; 3 Institute of Medical Biology, UiT The Arctic University of Norway, Tromso, Norway; 4 Department of Clinical Pathology, University Hospital of North Norway, Tromso, Norway; University of Bergen, NORWAY

## Abstract

**Background:**

Cancer Associated Fibroblasts (CAFs) are thought to regulate tumor growth and metastasis. Fibroblast Activating Protein 1 (FAP-1) is a marker for fibroblast activation and by many recognized as the main marker of CAFs. Alpha Smooth Muscle Actin (α-SMA) is a general myofibroblast marker, and can be used to identify CAFs. This study investigates the prognostic impact of FAP-1 and α-SMA in non-small cell lung cancer (NSCLC) patients and correlates their expression to 105 proteins investigated in the same cohort.

**Methods:**

Tumor specimens from 536 NSCLC patients were obtained and tissue micro-arrays were constructed. Immunohistochemistry was used to evaluate the expression of FAP-1 and α-SMA and explore their impact on survival and association with other tumor molecular markers in NSCLC patients.

**Results:**

High expression of FAP-1, but not α-SMA, in squamous cell carcinoma (SCC, P = 0.043, HR = 0.63 95% CI 0.40–0.99) was significantly associated with increased disease-specific survival. FAP-1 and α-SMA were not significantly correlated to each other. Analyses of FAP-1 and α-SMA associated with other tumor-related proteins revealed histotype-specific correlation patterns.

**Conclusion:**

The presence of FAP-1 expressing CAFs is an indicator of positive outcome for NSCLC-SCC patients. In addition, correlation analyses suggest FAP-1 and α-SMA to label different subsets of fibroblasts and their associations with other tumor-related proteins diverge according to histological subtype.

## Introduction

Non-small cell lung cancer (NSCLC) is one of the leading causes of cancer associated death worldwide, with both high incidence and mortality rates [1]. Current treatment strategies consist of wide tumor resection with the supplement of radio- and/or chemotherapy for subgroups of patients with poor prognosis [2]. NSCLC is a heterogeneous disease, including squamous cell carcinoma (SCC) and adenocarcinoma (ADC) as the dominant histological subgroups, which treatment-wise used to be considered a single entity. However, emerging evidence from molecular marker analyses indicate the molecular background of NSCLC subtypes to differ [3]. Extensive analyses of genetic alterations have revealed distinct NSCLC subtypes that can be targeted with modern therapeutic approaches. However, these new and exciting strategies have yet to be implemented in an adjuvant setting to improve the overall survival of NSCLC patients [3].

Over the last two decades, an increasing awareness of cancer as a complex and heterogenous disease has risen. This fact has shifted the focus of cancer research from a reductionist view including only malignant cells to include also the supportive stromal tissue surrounding the tumor. For colorectal, breast and lung cancer the prognostic impact of immune cells infiltrating the intratumoral stroma is thought to supplement and even surpass the established TNM staging algorithms [4–6]. Another fundamental player in the stromal compartment is the cancer associated fibroblasts (CAFs). Fibroblasts comprise a very heterogeneous and multi-functional cell population, which plays important regulatory roles in wound healing, embryonic development and cancer initiation and propagation [7]. In cancer, numerous studies have documented the importance of reciprocal interactions between malignant cells and CAFs [7]. In addition, CAFs regulate important elements in the tumor associated stroma including ECM, angiogenesis, recruitment and activation of immune cells and the recruitment of peripheral progenitor cells [7,8]. The generic name “CAF” encompasses a diverse set of cells that may have different origins and functions. Thus, the term "CAFs" may be referring to tumor resident fibroblasts, activated fibroblast, peritumoral fibroblasts, myofibroblasts, pericytes and other mesenchymal cells derived from circulating progenitors or after epithelial or endothelial trans-differentiation. Traditionally, CAFs have been identified by their wide-spread expression of alpha-smooth muscle actin (α-SMA), however, due to the notable cellular heterogeneity; α-SMA expression alone will not identify all CAFs [7,8]. Hence, other CAF markers in use are vimentin, fibronectin, fibroblast-specific protein 1 (FSP-1), fibroblast activation protein 1 (FAP-1) and PDGFR-α/β [8,9]. FAP-1 is a membrane-anchored serine protease, which is selectively expressed by stromal and mesenchymal cells during wound healing, fibrotic reactions, inflammatory conditions and tumor development [8]. Because of the transient expression of this marker during activation, FAP-1 has been used in many studies to identify activated tumor fibroblasts [8].

Several studies have associated the presence of different functional subsets of CAFs in primary NSCLC with adverse prognosis, including CAF^α-SMA^ and CAF^TGF-β^ in a cohort of mixed NSCLC [10] and CAF^Podoplanin^ in lung ADC [11–13] and SCC [14]. In addition, the presence of CAF^Podoplanin^ in lymph node metastases of nodal stage 2 lung SCC patients has been associated with a higher rate of mediastinal recurrence [15,16]. Only one small study (n = 59) has investigated the presence of CAF^FAP^ in NSCLC and found an association with worse prognosis [17].

We have previously evaluated 105 molecular tumor-related proteins in a cohort of unselected resected stage I-IIIA NSCLC patients. The aim of this study was to investigate the prognostic impact of CAF^FAP^ and CAF^α-SMA^ in NSCLC, and to correlate their presence with the expression of other tumor molecular markers.

## Material and Methods

### 1. Patients and Clinical Samples

Primary tumor tissue from 536 patients diagnosed with, and surgically resected for stage I-IIIA NSCLC at the University Hospital of North-Norway and Nordland Central Hospital from 1990 through 2010 were included in this study. This represents an expansion and update of a previously existing cohort including patients with the same criteria from 1990 through 2004 [18]. The included patients were staged according to the 7th edition of the UICC TNM classification and classified according to the new pathological classification of lung cancer [19]. In total 633 patients were registered from the hospital databases. Of these 97 were excluded from the study due to: radiotherapy or chemotherapy prior to surgery (n = 15), other malignancy within five years before NSCLC diagnosis (n = 39), inadequate paraffin-embedded fixed tissue blocks (n = 25) and adenocarcinoma *in situ* (AIS), prior to 2011 classified as bronchoalveolar carcinoma *in situ* (BAC) < 3 cm on revision (n = 18). Thus 536 with complete medical records and available formalin-fixed paraffin-embedded (FFPE) tissue blocks were eligible.

This report includes follow-up data as of October 1^st^ 2013. The median follow-up of survivors was 73 months (range 0–267).

### 2. Micro-array Construction

All patient samples were reviewed independently by two pathologists. The most representative areas, containing both tumor and intratumoral stroma, were marked on the hematoxylin and eosin (H/E) slides and sampled for the tissue micro-array (TMA) blocks. The TMAs were assembled using a tissue-arraying instrument (Beecher Instruments, Silver Springs, MD, USA). The detailed methodology has been reported previously [20]. Briefly, we used a 0.6 mm stylet to sample four replicate cores from each of the study specimens.

To include all core samples, 12 TMA blocks were constructed. Multiple 4-μm sections were cut with a Micron microtome (HM355S) and stained by specific antibodies for immunohistochemistry (IHC) analysis.

### 3. Immunohistochemistry

Immunohistochemistry (IHC) assays for FAP-1were performed on the Ventana Discovery-Ultra automated immunostainer (Ventana Medical Systems, Tucson, AZ). Deparaffinization and on-board antigen retrieval were performed for 24 minutes at approximately 100°C with CC1 reagent, which is an EDTA-based proprietary Ventana solution (pH 8.0–8.5). FAP-1anti-rabbit polyclonal antibody (Ab53066, Abcam, dilution 1/50) was applied and incubated for 32 minutes. Slides were developed using Ultramap anti-rabbit HRP (Cat#760–4315, Ventana) and were detected using ChromoMap DAB (Cat#760–159, Ventana).

IHC assays for α-SMA were stained on Ventana Benchmark-ultra (Ventana Medical Systems Inc.). Epitope retrieval was accomplished with CC1 solution for 8 minutes. The α-SMA mouse monoclonal antibody (cat#760–2833, Ventana, clone 1A4) was incubated at 37° for 32 minutes and detected by using the Ultraview DAB Detection kit. Finally, to visualize the nuclei, slides were counterstained with Ventana Hematoxylin II reagent for 8 minutes, followed by a Bluing reagent for 4 minutes.

Two different controls for our staining method were applied. Firstly, control staining of the sections with an isotype-matched control antibody without the primary antibody. Secondly, multiple organ tissue micro-array as positive and negative tissue controls were used to verify the specificity of the staining in every staining procedure. The positive tissue controls comprised normal liver and breast carcinoma for FAP-1 and appendix and vessel wall for alpha-SMA; Negative tissue controls were samples of normal pancreas, lung, breast, tonsil and kidney for both Fap and α-SMA.

### 4. Antibody validation

**Fig 1:**
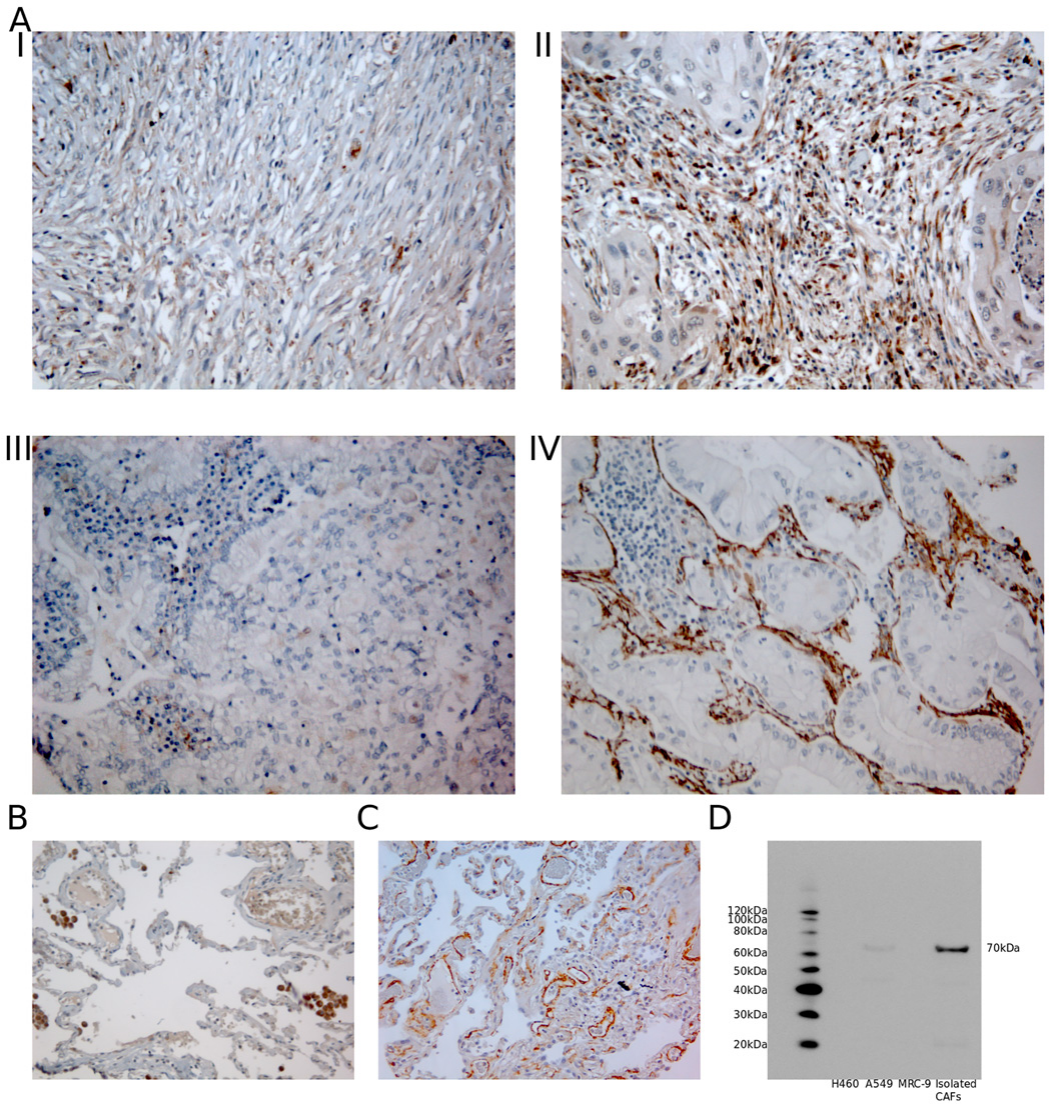
A) IHC analysis of TMA cores from NSCLC patients representing different stromal cell scores for expression of FAP-1 and α-SMA. I) Low stromal FAP-1; II) High stromal FAP-1; III) Low stromal α-SMA; IV) High stromal α-SMA. B) TMA control of healthy lung tissue showing strong FAP-1 positivity in normal lung macrophages, C) TMA control of healthy lung tissue showing some weak to intermediate α-SMA staining, D) Western blot for FAP-1 showing one clear 70 kDa band. Abbreviations: FAP-1, Fibroblast activating protein; α-SMA, alpha-smooth muscle actin.

Mouse-monoclonal anti-human α-SMA antibody was purchased from Ventana (cat#760–2833, Ventana, clone 1A4). This antibody has been subjected to extensive quality-control and validation procedures by the manufacturer which ensures proper and specific staining of the antigen in paraffin-embedded tissue. Polyclonal rabbit anti-human FAP-1 antibody was purchased from abcam (Cat.#:ab53066). Specificity test of this antibody was done in-house by western blot. Antibody was tested against whole cell extracts from several CAF cell lines and from the lung tumor cell line A549. Western blots showed a single band of about 70 KDa in protein extracts from CAFs (Fig 1D). This antibody has previously been tested on isolated CAFs by IHC [21].

### 5. Scoring of Immunohistochemistry

Representative and viable tissue sections were reviewed using a (Leica DM 2500" Leica Microsystems Ltd., CH9435 Heerbrugg, Switzerland). Based on initial review, author Al-Saad established a semi-quantitative score for each marker. The slides were scored for FAP-1 in the total material and α-SMA in the updated material (cases from 2005 through 2010) by authors Rakaee Khanehkenari and Martinez. α-SMA had previously been scored for cases from 1990 through 2004. The dominant staining intensity in percentage of positive cells in the intraepithelial stromal compartment was scored as follows: 0 = no staining, 1 = 1–10%, 2 = 11–50% and 3 = > 50%. When assessing a given core, the observers were blinded to the scores of other variables and to outcome. We did not observe any staining of tumor epithelial cells for either α-SMA nor FAP-1. This is is similar to reports from other studies [22].

High expression was defined as the optimal cut-off point discriminating the high/low groups according to survival. High expression was defined as a score > 0.5 (FAP-1) and >2 (α-SMA).

### 6. Statistical Methods

All statistical analyses were conducted using RStudio 0.98.1091 with R version 3.1.1.1[23] and libraries "survival"[24], "car"[25], "ggplot2"[26], "gridExtra"[27], "Hmisc"[28] and "irr"[29].

The IHC scores from each observer were compared for interobserver reliability using a two-way random effects model with absolute agreement definition and Cohens kappa-statistics with equal weights. The intraclass correlation coefficient (reliability coefficient) and Cohen's kappa were obtained from these results.

The Chi-square and Fischer's Exact tests were used to examine the association between molecular marker expression and clinipathological variables and to check whether there were differences in marker expression in the original and updated patient cohort. Spearman`s rank-correlation was used to examine the associations between marker expressions. Due to the large number of correlation analyses, Bonferroni corrections for P-values were conducted for these analyses.

Univariate survival analyses were done using the Kaplan-Meier method. Statistical differences between survival curves were assessed by the log-rank test. Disease-specific survival (DSS) was defined as the time from surgery to lung cancer related death. Multivariate analysis, using the Cox proportional hazards model, was carried out to assess the independent value of pretreatment variables in the presence of other variables. Only variables with P < 0.25 from the univariate analyses or otherwise thought to be important were explored in the Cox regression analysis.

The significance level used for survival analyses was P < 0.05

### 7. Ethical Clearance

The study was approved by the Regional Committee for Medical and Health Research Ethics (Northern Norway, UNN: protocol ID: 2011/2503) and the need for consent was waived unnecessary. The collection and storing of the clinical database was approved by the National Data Inspection Board. The reporting of clinicopathological variables, survival data and biomarker expression was conducted in accordance with the REMARK guidelines.

## Results

### 1. Clinicopathological Variables

Clinicopathological variables are summarized in Table 1. Median age at time of diagnosis was 67.2 years and 32% of the patients were female. The histological distribution of the 536 cases were 289 squamous cell carcinomas (SCC), 201 adenocarcinomas (ADC) and 46 undifferentiated carcinomas (NOS). In addition to surgical resection, 64, 89 and 12 patients received adjuvant radiotherapy, chemotherapy and radiochemotherapy, respectively.

**Table 1:** Prognostic clinicopathological and FAP-1 and α-SMA as predictors of disease-specific survival in resected non-small-cell lung cancer patients and in subgroups with squamous cell carcinoma and adenocarcinoma (univariate analyses, log-rank test, n = 535, 289 and 201 respectively).

	All					SCC					ADC				
	N(%)	5 Year	Median	HR(95%CI)	P	N(%)	5 Year	Median	HR(95%CI)	P	N(%)	5 Year	Median	HR(95%CI)	P
Age					0.711					0.654					0.505
≤65	227(42)	57	127	1		106(37)	64	235	1		102(51)	48	54	1	
>65	309(58)	58	NA	0.95(0.73–1.24)		183(63)	66	NA	0.91(0.61–1.36)		99(49)	49	57	0.87(0.59–1.3)	
Gender					**0.026**					0.108					**0.050**
Female	170(32)	63	190	1		73(25)	73	NA	1		83(41)	56	190	1	
Male	366(68)	55	88	1.4(1.06–1.84)		216(75)	63	235	1.49(0.96–2.31)		118(59)	43	51	1.5(1.01–2.23)	
ECOG					**0.015**					0.158					**0.003**
0	310(58)	62	235	1		158(55)	69	235	1		122(61)	56	NA	1	
1	190(35)	52	71	1.45(1.09–1.93)		110(38)	61	114	1.47(0.97–2.23)		67(33)	40	50	1.57(1.02–2.4)	
2	36(7)	48	36	1.61(0.83–3.09)		21(7)	67	NA	1.08(0.45–2.6)		12(6)	17	25	3.25(0.96–11.03)	
Smoking					**0.039**					0.19					0.68
Previous	17(3)	44	20	1		7(2)	50	19	1		9(4)	44	21	1	
Previous	342(64)	62	235	0.56(0.25–1.24)		182(63)	69	235	0.58(0.14–2.37)		125(62)	50	68	0.69(0.26–1.84)	
Present	177(33)	51	71	0.75(0.33–1.7)		100(35)	60	114	0.82(0.2–3.41)		67(33)	45	57	0.73(0.27–1.99)	
Weightloss					0.961					0.689					0.536
<10%	480(90)	58	127	1		256(89)	66	235	1		184(92)	49	57	1	
≥10%	55(10)	59	NA	0.99(0.63–1.56)		32(11)	62	NA	1.14(0.57–2.28)		17(8)	40	47	1.24(0.59–2.63)	
Missing	1(0)					1(0)									
Surgical procedure					**<0.001**					**<0.001**					**<0.001**
Wedge/Lobectomy	394(74)	63	190	1		197(68)	72	235	1		161(80)	54	104	1	
Pulmonectomy	142(26)	42	30	1.98(1.43–2.74)		92(32)	50	35	1.99(1.28–3.09)		40(20)	25	24	2.66(1.46–4.84)	
Margins					0.129					0.252					0.018
Free	489(91)	59	190	1		257(89)	67	235	1		189(94)	50	68	1	
Not free	47(9)	47	57	1.39(0.85–2.29)		32(11)	57	114	1.39(0.73–2.63)		12(6)	0	35	2.33(0.81–6.69)	
Tstage					**<0.001**					**<0.001**					**<0.001**
I	168(31)	72	235	1		83(29)	78	235	1		74(37)	67	190	1	
II	265(49)	57	91	1.74(1.3–2.32)		147(51)	66	NA	1.88(1.22–2.89)		94(47)	43	47	1.94(1.27–2.95)	
III	97(18)	36	30	2.84(1.87–4.31)		56(19)	46	33	2.93(1.62–5.31)		31(15)	16	25	3.48(1.76–6.9)	
IV	6(1)	20	15	4.89(0.89–26.9)		3(1)	0	10	17.41(0.22–1371.77)		2(1)	50	13	1.76(0.23–13.27)	
Nstage					**<0.001**					**<0.001**					**<0.001**
0	364(68)	69	235	1		198(69)	77	235	1		133(66)	60	190	1	
1	118(22)	36	35	2.76(1.93–3.94)		73(25)	45	35	3.26(1.99–5.35)		39(19)	25	30	2.41(1.38–4.2)	
2	54(10)	21	19	4.23(2.43–7.37)		18(6)	18	13	7.12(2.44–20.77)		29(14)	23	24	2.88(1.42–5.82)	
Pstage					**<0.001**					**<0.001**					**<0.001**
I	256(48)	72	235	1		127(44)	82	235	1		105(52)	65	190	1	
II	194(36)	53	84	1.89(1.42–2.51)		126(44)	60	114	2.5(1.66–3.77)		56(28)	34	43	2.07(1.3–3.28)	
IIIA	86(16)	20	17	4.58(2.87–7.32)		36(12)	23	15	7.15(3.23–15.84)		40(20)	16	24	3.37(1.8–6.33)	
Histology					**0.040**										
SCC	289(54)	65	235	1											
ADC	201(38)	48	57	1.43(1.08–1.89)											
NOS	46(9)	50	83	1.29(0.8–2.08)											
Differentiation					**<0.001**					**0.033**					**0.006**
Poor	231(43)	49	51	1		104(36)	57	84	1		81(40)	38	43	1	
Moderate	240(45)	63	190	0.67(0.5–0.89)		155(54)	70	235	0.63(0.41–0.97)		85(42)	50	68	0.69(0.44–1.07)	
Well	65(12)	70	NA	0.44(0.29–0.66)		30(10)	72	NA	0.47(0.24–0.94)		35(17)	69	NA	0.36(0.21–0.63)	
Vascular infiltration					**<0.001**					**0.029**					**0.012**
No	437(82)	62	235	1		231(80)	69	235	1		172(86)	52	71	1	
Yes	97(18)	38	35	1.89(1.29–2.78)		58(20)	53	71	1.65(0.97–2.82)		27(13)	26	27	1.9(1–3.62)	
Missing	2(0)										2(1)				
FAP-1					0.070					**0.043**					0.986
Low	98(18)	51	71	1		52(18)	54	71	1		40(20)	52	71	1	
High	419(78)	59	235	0.75(0.54–1.03)		229(79)	69	235	0.63(0.4–0.99)		154(77)	47	54	1(0.62–1.63)	
Missing	19(4)					8(3)					7(3)				
α-SMA					0.597					0.220					0.797
Low	362(68)	57	127	1		198(69)	64	235	1		137(68)	48	57	1	
High	173(32)	59	NA	0.93(0.7–1.23)		90(31)	69	NA	0.75(0.49–1.16)		64(32)	48	54	1.06(0.69–1.61)	
Missing	1(0)					1(0)									

Abbreviations: SCC, squamous-cell carcinoma; ADC, adenocarcinoma; NOS, Not otherwise specified; FAP-1, Fibroblast activating protein; α-SMA, alpha-smooth muscle actin.

### 2. Interobserver Reliability and Tests for Differences in Expression

The intraclass correlation coefficients and Cohen's kappa were 0.759 (P < 0.001) and 0.598 (P < 0.001) for FAP-1 and 0.790 (P < 0.001) and 0.657 (P < 0.001) for α-SMA. No difference in expression for FAP-1 (P = 0.284) or α-SMA (P = 1.000) was observed according to time period (original vs updated cohort).

### 3. Survival Analyses

**Fig 2:**
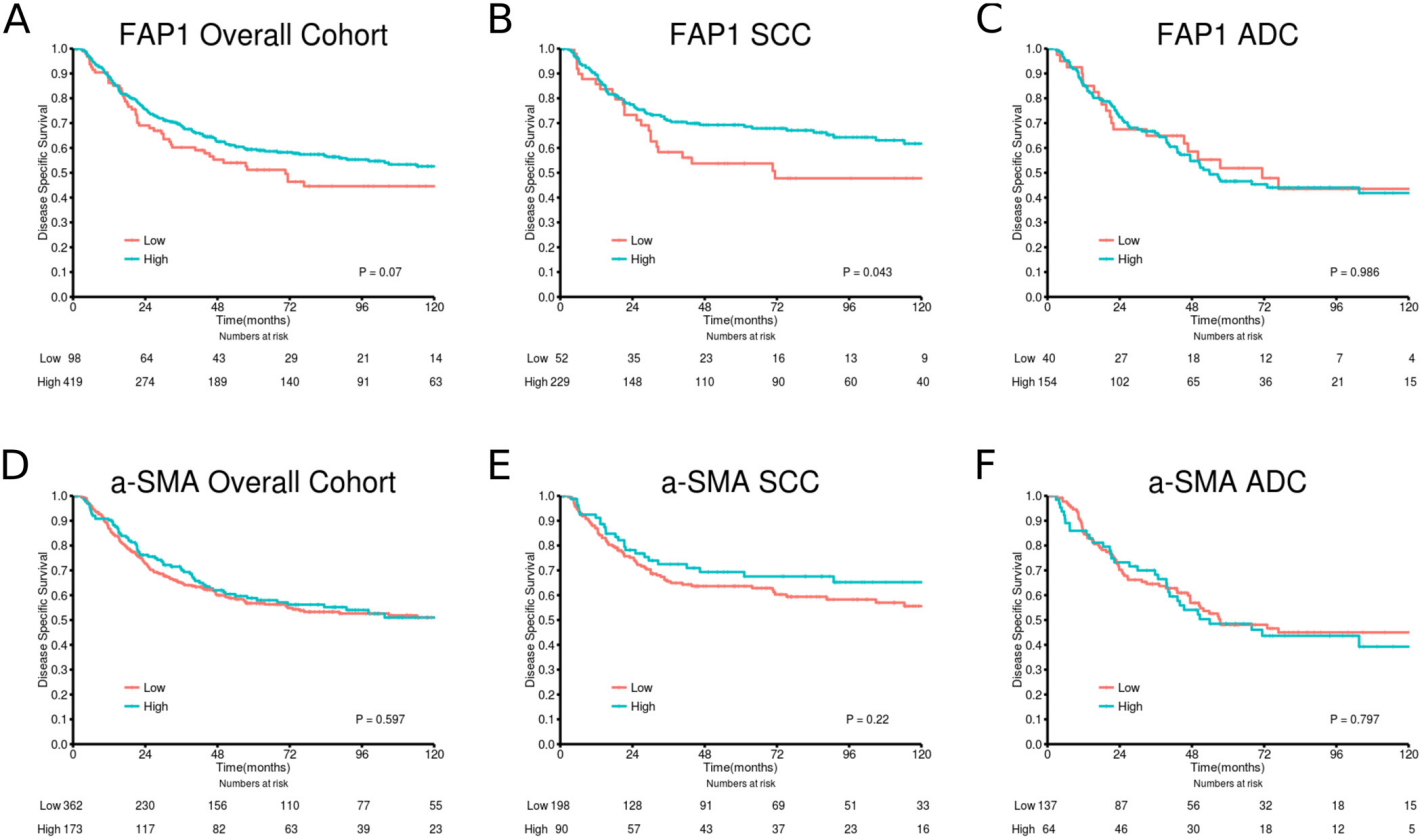
Disease-specific survival curves for A) FAP-1 in the overall cohort; B) FAP-1 in SCC; C) FAP-1 in ADC; D) α-SMA in the overall cohort; E) α-SMA in SCC; F) α-SMA in ADC. Abbreviations:SCC, squamous-cell carcinoma; ADC, adenocarcinoma; FAP-1, Fibroblast activating protein; α-SMA, alpha-smooth muscle actin.

Table 1 summarizes the impact of clinicopathological variables and investigated markers in the overall group and stratified into histological subgroups. Fig 2 panels A-F shows the survival curves of FAP-1 and α-SMA low *vs* high expression in the overall cohort and stratified into SCC and ADC subgroups. High expression of FAP-1 was a significant positive marker for survival in the SCC subgroup (P = 0.043). Neither FAP-1, nor α-SMA expression in tumor stroma were significant independent variables in multivariate analyses.

### 4. Expression of FAP-1 and α-SMA and their Correlations

FAP-1 and α-SMA expression was localized in the cytoplasm of stromal cells (Fig 1A). Of note, FAP-1 expression was not restricted to fibroblast-like cells as intratumoral and healthy lung tissue macrophages were strongly stained by the antibody (Fig 1B). However, when assessing the cores, macrophage expression of FAP-1 was not taken into consideration. S1 Table summarizes the associations of FAP-1 and α-SMA with clinicopathological markers. After corrections for multiple testing no significant associations were observed.

Table 2 summarizes the significant correlations of FAP-1 and α-SMA with tumor-related proteins previously analyzed in this patient cohort. S2 Table names all markers included in the correlation analyses (n = 105). After corrections for multiple testing, significant correlations were observed between FAP-1 and the following markers: stromal expression of HIF2 (r = 0.26), LDH5 (r = 0.25), FOXP3 (r = 0.23), CSF1R (r = 0.26) and miR21 (r = 0.27) and tumor cell expression of p21 (r = 0.30) in the total cohort; stromal expression of CSF1R (r = 0.29) and mir21 (r = 0.31) in the SCC subgroup; stromal expression of HIF2 (r = 0.42) and tumor cell expression of MCT3 (r = -0.42) in the ADC subgroup. α-SMA showed no significant correlations, neither in the whole material, nor in subgroups, after corrections for multiple testing was conducted.

**Table 2:** Significant Spearman rank-correlations with R-values > 0.20 between tumor and stromal cell expression of FAP-1 or α-SMA in TMA samples from NSCLC patients (n = 536 for correlations with CD3 and correlations between FAP-1 and α-SMA and 326 for the remaining correlations) and in subgroup analyses of NSCLC according to histology (SCC n = 289 for correlations with CD3 and correlations between FAP-1 and α-SMA and 191 for the remaining correlations, ADC N = 201 for correlations with CD3 and correlations between FAP-1 and α-SMA and 95 for the remaining correlations).

	All cases		SCC		ADC		
	FAP-1	α-SMA	FAP-1	α-SMA	FAP-1	α-SMA	
	**Correlations according to marker expressions in intratumoral stroma**						
Ang2			0.21**				Ang
bFGF				-0.21**			Ang
PDGF-D			0.23**				Ang
PDGFRalpha			0.2**				Ang
PDGFRbeta						0.21*	Ang
CXCL16	0.21***		0.26***				Ch
DLL4					0.26*		Ch
bAkt						0.21*	EMT
cAkt	0.21***				0.21*		EMT
ERK3			0.28***		-0.21*		EMT
Igf1			0.21**				EMT
Masson's trichrome		0.2***		0.26***			EMT
NfκB			0.22**			0.24*	EMT
HIF2	**0.26** #		0.22**		**0.42** #		Hyp
LDH5	**0.25** #		0.25***				Hyp
CD3					0.21**		Imad
FOXP3	**0.23#**				0.23*		Imad
CD138					0.23*		Imin
CD56					0.21*		Imin
Cox	0.21***				0.23*		Imin
CSF1R	**0.26** #		**0.29** #				Imin
MCT1	0.21***		0.22**		0.27*		Met
MCT2					-0.25*		Met
MCT4					0.26*		Met
miR21	**0.27** #		**0.31** #				miR
miR210			0.21**				miR
Igfbp					-0.3**		other
Ki67				-0.22**	0.28*		other
Notch4					0.25*		other
Par6			0.21**				other
	**Correlations according to marker expressions in tumor cell nests**						
ANG4						0.25*	Ang
VEGF-C					-0.22*		Ang
VEGFR2					-0.32**		Ang
Vessel co option						0.21*	Ang
Bad in cytoplasm			-0.21**				Ap
Bad in nucleus			-0.22**				Ap
p21	**0.3** #		0.27***		0.29**		EMT
sAkt					0.23*		EMT
SKB2				-0.2**			EMT
PHD3						0.21*	Hyp
CD3						0.21**	Imad
CD1A			0.21**				Imin
CSF1R					0.22*		Imin
MCT2					0.27*		Met
MCT3					**-0.42** #		Met
miR21	0.22***		0.26***				miR
miR182b	0.2***				0.25*		miR
Ki67					0.25*		other

Abbreviations: SCC, squamous-cell carcinoma; ADC, adenocarcinoma; FAP-1, Fibroblast activating protein; α-SMA, alpha-smooth muscle actin; Ang, Angiogenin; bFGF, basic fibroblast growth factor; PDGF, platelet-derived growth factor; PDGFR, platelet-derived growth factor receptor; CXCL, C-X-C motif ligand; DLL, Delta-Like; ERK, extra-cellular signal regulated kinase; Igf; insulin growth factor; NfκB, nuclear factor kappa-light-chain-enhancer of activated B cells; HIF, hypoxia-induced factor; LDH, lactate dehydrogenase; CD, cluster of differentiation; FOXP3; forkhead box P3; Cox, cyclo-oxygenase; CSF1R, colony-stimulating factor 1 receptor; MCT, monocarboxylate transporter; miR, micro RNA; Igfbp, insulin growth factor binding protein; Par, partitioning-defective; VEGF, vascular endothelial growth factor; VEGFR, vascular endothelial growth factor receptor; Bad, BCL-2 associated death promoter; p21, protein 21; SKB,SHK1 binding protein; PHD, prolyl hydroxylase-domain.

*significant at p > 0.05,

**significant at p > 0.01,

***significant at p > 0.001,

^#^ significant after Bonferroni correction for multiple tests

## Discussion

The present study was initiated to explore the potential prognostic impact of CAFs in NSCLC, and the existing correlations of these cells with other relevant tumor biological markers. In a recent review, Cortez et al. highlighted four functional CAF subsets according to expression of effector molecules and receptors; CAF^FAP^, CAF^FSP1^, CAF^PDGFR-α^ and CAF^PDGFR-β^ [8]. Significant efforts have been devoted to explore the role of CAF subtypes in NSCLC. The most studied subtype, CAF^Podoplanin^, has been associated with shorter overall survival for both ADC and SCC lung cancer patients [11–14] and their presence in mediastinal lymph-node metastases increases the risk of mediastinal recurrence [15,16]. Due to CAF heterogeneity and the lack of a single universal marker, we chose to identify CAFs by two of the most widely used markers that exclude quiescent fibroblasts from CAFs, namely FAP-1 and α-SMA. In contrast to previous reports on CAFs and NSCLC, we find that CAF^FAP^ are predictors of favorable prognosis in the SCC subgroup. Intriguingly, in our study FAP-1 and α-SMA expression do not correlate with each other, indicating that these markers identify different CAFs subtypes. Lastly, our study uncovers interesting correlations between CAF^FAP^ and other tumor molecular markers that aid in understanding the mechanisms behind the observed impact in prognosis. To our knowledge this study represents the largest evaluation of CAF^FAP^ and CAF^α-SMA^ in NSCLC.

### 1. CAF^FAP^ in NSCLC

Liao et al. explored the role of CAF^FAP^ in a small series of 59 NSCLC patients and found higher levels of FAP expressing stromal cells to be an independent indicator of poor overall survival [17]. In contrast, we found, in a larger patient cohort, the presence of CAF^FAP^ to predict increased disease-specific survival in SCC, but not in ADC patients. The defined role of CAF^FAP^ is poorly understood and their presence has been investigated in other tumor groups with differing results. In invasive ductal carcinoma of the breast, CAF^FAP^ are associated with increased survival, while in ADC of the pancreas and rectum they are associated with decreased survival [30–32]. These results, in line with our study, indicate that CAF^FAP^ interact with tumor cells and other players in the tumor micro-environment in differential ways according to the context, tumor stage and tissue of origin. The difference between our study and the Liao study could be explained by their inferior number of included patients, the use of whole-slide examinations *vs* TMA core samples, or differences in the patient cohorts otherwise unaccounted for.

Obviously, our TMA based study cannot directly explain the mechanisms of these observed effects. Nevertheless, extensive correlation analyses (Table 2) in our patient cohort reveal strong positive associations between CAF^FAP^ and stromal expression of colony-stimulating factor receptor 1 (CSF1R) and micro-RNA 21 (miR21) in SCC and stromal expression of hypoxia-induced factor 2 (HIF2) in ADC and strong negative association with tumor cell expression of monocarboxylate transporter 3 (MCT3) in ADC. Stromal CSF1R could be expressed both in macrophages and in myeloid dendritic cells (DC) [33]. The presence of type M1 cytotoxic macrophages could explain the survival benefit we observe in CAF^FAP^ SCC patients, by macrophages directly targeting tumor cells. CSF1R positive DCs are able to take up antigen and their presence may indicate a local immune-response [33]. miR21 is generally described as an onco-miR and is expressed in most solid cancers [34]. A recent study has established a link between miR21 expression and CAF formations and could explain the positive correlation seen in our cohort of SCC patients [35]. We have previously identified high stromal miR21 expression to predict poor survival for a subset of NSCLC patients [36]. In this study the low miR21 expressing group comprised only 21 patients which might possibly explain the observed increased survival in the SCC subgroup, even though a moderately strong correlation with miR21 is observed.

Previous results have shown high stromal expression of HIF2 to be a favorable indicator of prognosis of SCC, but not ADC, while tumor cell expression of MCT3 was unrelated to prognosis regardless of histological subgroups [37,38].

Strategies have been developed to utilize the presence FAP-expressing stromal cells in the treatment of cancer. These can be divided into two categories i) those that targets and neutralizes CAF^FAP^ directly and ii) those that utilize FAP expression in tumor stromal cells to deliver a drug to the tumor site [39–43]. Our results indicate that for NSCLC patients, and especially in the SCC subgroup, the first approach might prove detrimental.

### 2. CAF^α-SMA^ in NSCLC

α-SMA is generally accepted as a myofibroblast marker, although the labeling is unspecific and other cell-types such as pericytes may also be stained [8]. Chen et al. explored the roles of CAF^α-SMA^ and CAF^TGF-β^ in a small cohort of 78 NSCLC patients of all stages and histologies using whole tumor tissue slides. They found CAF^α-SMA^ to be an independent indicator of adverse outcome [10]. In our study, the presence of CAF^α-SMA^ did not show any prognostic information, neither in the overall population, nor in subpopulations according to histology. The difference in outcome could be explained by the different approaches used (whole slide vs TMA) or by the different numbers of patients in the cohorts. Furthermore, the etiology of the lung cancer cases could also be different. Assuming that Chen et al. mainly included patients from the Suzhou region, which is a densely populated and highly industrialized area, it is conceivable that the study included a larger number of patient with smoking-unrelated lung cancer. Unfortunately, the Chen study did not include information about smoking status. In contrast, our study recruited patients from Northern-Norway, a sparsely populated region with hardly any polluting industry, and only 18 out of 536 of the included patients had never smoked. It would be interesting to explore how CAF^α-SMA^ reacts to different stimuli (eg. smoking *vs* industrial pollution).

Of interest, no significant correlations (after correction for multiple testing) were observed between the presence of CAF^α-SMA^ and other tumor-related proteins previously investigated in our patient cohort.

## Conclusion

In NSCLC, our results indicate that the presence and prognostic significance of CAF subtypes differ between histological subgroups and that the presence of CAF^FAP^ may be beneficial for the survival of NSCLC SCC patients. These results are in contrast to one other small study of CAF^FAP^ in NSCLC. This discrepancy is important as CAF^FAP^targeting strategies are being developed. The utilization of such strategies in NSCLC patients, especially in the SCC subgroup, should be approached with diligence until these results are further validated and explored in *in-vitro* and in *in-vivo* tumor models of both SCC and ADC NSCLC.

## Supporting Information

S1 Archive Compressed version of S1 and S2 Tables. (ZIP)

S1 Table Correlations between FAP-1 and α-SMA and clinicopathological variables. (DOCX)

S2 Table List of markers investigated in our cohort. (ODS)
