# Supplementary material for: Cancer Associated Fibroblasts in Stage I-IIIA NSCLC: Prognostic Impact and Their Correlations with Tumor Molecular Markers
Source: PLoS One. 2015 Aug 7;10(8):e0134965. doi: 10.1371/journal.pone.0134965 (PMC4529239; doi:10.1371/journal.pone.0134965)
Supplement: S1 Table — (DOCX) [file pone.0134965.s002.docx]

**Supplemental table 1:** Associations between FAP1 and α-SMA and clinicopathological markers in the overall cohort and in the SCC and ADC subgroups. Chi-square and Fishers exact tests.

|  | ALL |  |  |  |  |  | SCC |  |  |  |  |  | ADC |  |  |  |  |  |
| --- | --- | --- | --- | --- | --- | --- | --- | --- | --- | --- | --- | --- | --- | --- | --- | --- | --- | --- |
|  | FAP1 | |  | α-SMA | |  | FAP1 | |  | α-SMA | |  | FAP1 | |  | α-SMA | |  |
|  | Low | High | P | Low | High | P | Low | High | P | Low | High | P | Low | High | P | Low | High | P |
| Age |  |  | 0.051 |  |  | 0.722 |  |  | 0.330 |  |  | 0.977 |  |  | 0.026 |  |  | 0.104 |
| ≤65 | 51 | 170 |  | 156 | 71 |  | 23 | 82 |  | 73 | 33 |  | 27 | 71 |  | 71 | 31 |  |
| >65 | 47 | 249 |  | 206 | 102 |  | 29 | 147 |  | 127 | 55 |  | 13 | 83 |  | 57 | 42 |  |
| Gender |  |  | 0.625 |  |  | 0.195 |  |  | 1.000 |  |  | 0.410 |  |  | 0.277 |  |  | 0.028 |
| Female | 34 | 132 |  | 108 | 62 |  | 13 | 59 |  | 54 | 19 |  | 20 | 60 |  | 45 | 38 |  |
| Male | 64 | 287 |  | 254 | 111 |  | 39 | 170 |  | 146 | 69 |  | 20 | 94 |  | 83 | 35 |  |
| ECOG |  |  | 0.266 |  |  | 0.186 |  |  | 0.182 |  |  | 0.415 |  |  | 0.734 |  |  | 0.117 |
| 0 | 60 | 240 |  | 208 | 102 |  | 27 | 127 |  | 112 | 46 |  | 27 | 92 |  | 73 | 49 |  |
| 1 | 35 | 147 |  | 134 | 66 |  | 24 | 83 |  | 76 | 33 |  | 11 | 52 |  | 49 | 18 |  |
| 2 | 3 | 32 |  | 20 | 16 |  | 1 | 19 |  | 12 | 9 |  | 2 | 10 |  | 6 | 6 |  |
| Smoking |  |  | 0.003 |  |  | 0.412 |  |  | 0.222 |  |  | 0.619 |  |  | 0.003 |  |  | 0.305 |
| Never | 7 | 9 |  | 12 | 5 |  | 2 | 4 |  | 4 | 3 |  | 5 | 4 |  | 7 | 2 |  |
| Previous | 41 | 130 |  | 113 | 64 |  | 21 | 75 |  | 68 | 32 |  | 18 | 47 |  | 38 | 29 |  |
| Present | 50 | 280 |  | 237 | 104 |  | 29 | 150 |  | 128 | 53 |  | 17 | 103 |  | 83 | 42 |  |
| Weigthhloss |  |  | 0.035 |  |  | 0.836 |  |  | 0.087 |  |  | 1.000 |  |  | 0.532 |  |  | 1.000 |
| <10% | 94 | 368 |  | 325 | 154 |  | 50 | 198 |  | 177 | 78 |  | 38 | 140 |  | 117 | 67 |  |
| ≥10% | 4 | 50 |  | 36 | 19 |  | 2 | 30 |  | 22 | 10 |  | 2 | 14 |  | 11 | 6 |  |
| Surgical procedure |  |  | 0.132 |  |  | 0.078 |  |  | 0.349 |  |  | 0.070 |  |  | 0.323 |  |  | 0.456 |
| Wedge/Lobectomy | 65 | 312 |  | 257 | 136 |  | 32 | 159 |  | 129 | 67 |  | 29 | 125 |  | 100 | 61 |  |
| Pulmonectomy | 33 | 107 |  | 105 | 37 |  | 20 | 70 |  | 71 | 21 |  | 11 | 29 |  | 28 | 12 |  |
| Margins |  |  | 0.991 |  |  | 0.922 |  |  | 0.620 |  |  | 0.769 |  |  | 0.715 |  |  | 1.000 |
| Free | 90 | 382 |  | 331 | 157 |  | 48 | 203 |  | 179 | 77 |  | 37 | 145 |  | 120 | 69 |  |
| Not free | 8 | 37 |  | 31 | 16 |  | 4 | 26 |  | 21 | 11 |  | 3 | 9 |  | 8 | 4 |  |
| Tstage |  |  | 0.265 |  |  | 0.272 |  |  | 0.012 |  |  | 0.091 |  |  | 0.422 |  |  | 0.664 |
| I | 39 | 124 |  | 118 | 50 |  | 25 | 56 |  | 65 | 18 |  | 12 | 60 |  | 44 | 30 |  |
| II | 43 | 210 |  | 172 | 92 |  | 20 | 122 |  | 93 | 53 |  | 21 | 69 |  | 61 | 33 |  |
| III | 15 | 80 |  | 66 | 31 |  | 7 | 48 |  | 39 | 17 |  | 6 | 24 |  | 21 | 10 |  |
| IV | 1 | 5 |  | 6 | 0 |  | 0 | 3 |  | 3 | 0 |  | 1 | 1 |  | 2 | 0 |  |
| Nstage |  |  | 0.939 |  |  | 0.881 |  |  | 0.702 |  |  | 0.907 |  |  | 0.806 |  |  | 0.981 |
| 0 | 66 | 287 |  | 246 | 117 |  | 34 | 160 |  | 135 | 62 |  | 28 | 101 |  | 85 | 48 |  |
| 1 | 21 | 90 |  | 81 | 37 |  | 14 | 55 |  | 52 | 21 |  | 6 | 31 |  | 25 | 14 |  |
| 2 | 11 | 42 |  | 35 | 19 |  | 4 | 14 |  | 13 | 5 |  | 6 | 22 |  | 18 | 11 |  |
| Pstage |  |  | 0.434 |  |  | 0.701 |  |  | 0.553 |  |  | 0.498 |  |  | 1.000 |  |  | 0.854 |
| I | 50 | 196 |  | 175 | 80 |  | 25 | 99 |  | 90 | 36 |  | 21 | 80 |  | 66 | 39 |  |
| II | 30 | 157 |  | 127 | 67 |  | 19 | 102 |  | 83 | 43 |  | 11 | 44 |  | 35 | 21 |  |
| IIIA | 18 | 66 |  | 66 | 26 |  | 8 | 28 |  | 27 | 9 |  | 8 | 30 |  | 27 | 13 |  |
| Histology |  |  | 0.612 |  |  | 0.260 |  |  |  |  |  |  |  |  |  |  |  |  |
| SCC | 52 | 229 |  | 200 | 88 |  |  |  |  |  |  |  |  |  |  |  |  |  |
| ADC | 40 | 154 |  | 128 | 73 |  |  |  |  |  |  |  |  |  |  |  |  |  |
| LCC | 6 | 36 |  | 34 | 12 |  |  |  |  |  |  |  |  |  |  |  |  |  |
| Differentiation |  |  | 0.340 |  |  | 0.491 |  |  | 0.307 |  |  | 0.374 |  |  | 0.021 |  |  | 0.685 |
| Poor | 40 | 180 |  | 150 | 81 |  | 23 | 76 |  | 67 | 37 |  | 11 | 68 |  | 49 | 32 |  |
| Moderate | 42 | 193 |  | 166 | 73 |  | 25 | 127 |  | 111 | 43 |  | 17 | 66 |  | 55 | 30 |  |
| Well | 16 | 46 |  | 46 | 19 |  | 4 | 26 |  | 22 | 8 |  | 12 | 20 |  | 24 | 11 |  |
| Vascular infiltration |  |  | 0.775 |  |  | 0.874 |  |  | 0.662 |  |  | 0.058 |  |  | 1.000 |  |  | 0.123 |
| No | 77 | 344 |  | 294 | 143 |  | 40 | 189 |  | 154 | 77 |  | 33 | 132 |  | 113 | 59 |  |
| Yes | 19 | 75 |  | 66 | 30 |  | 12 | 44 |  | 46 | 11 |  | 5 | 22 |  | 13 | 14 |  |

Abbreviations: SCC, squamous-cell carcinoma; ADC, adenocarcinoma; LCC, large-cell undifferentiated carcinoma; FAP1, Fibroblast activating protein; α-SMA, alfa-smooth muscle actin
